# Factors Influencing Healthcare Engagement Among Low-Income Women Experiencing Headaches

**DOI:** 10.7759/cureus.102004

**Published:** 2026-01-21

**Authors:** Maithili Bhagat, Aaron Lorton, Sara Kalout, Sharon Casapulla, Abhishek Wajpe

**Affiliations:** 1 Medicine, Ohio University Heritage College of Osteopathic Medicine, Cleveland, USA; 2 Rural and Underserved Programs, Ohio University Heritage College of Osteopathic Medicine, Athens, USA; 3 Biostatistics, Ohio University Heritage College of Osteopathic Medicine, Athens, USA

**Keywords:** headache care, headache medicine, healthcare disparities, insurance barriers, low-income patients, medically underserved populations, migraine treatment, rural health disparities, utilization of healthcare, women’s health

## Abstract

Background

Headaches are a leading cause of disability among women, yet healthcare engagement in low-income populations remains poorly understood. Despite the high burden of disease, women with limited insurance coverage may delay or avoid seeking care due to barriers such as transportation difficulties, appointment wait times, and limited local providers. These challenges are often compounded in rural and medically underserved regions, where access to care is further reduced, and stigma or mistrust of the medical system may discourage care-seeking.

Objectives

This study aims to 1) assess care-seeking behaviors among Medicaid-enrolled or uninsured women with headache disorders and 2) identify demographic, geographic, and personal factors associated with barriers to care.

Methods

A cross-sectional survey was conducted among 156 Medicaid-enrolled or uninsured women recruited via community centers and email outreach to Ohio University-affiliated networks. Participants completed an online screening questionnaire to confirm insurance status and a novel seven-item Headache Severity and Affect Screening (HSAS) to assess headache burden. Eligible participants were surveyed on demographics, geographic location (categorized as rural, medically underserved area, or Appalachian), care-seeking behavior, and barriers to accessing care. Those who had sought treatment from a physician were asked about satisfaction with care and barriers; those who had not were asked about obstacles to care, such as stigma, transportation, cost, availability, language, mistrust, and perceived severity of headaches. Associations were assessed using chi-square and ANOVA.

Results

Perceived seriousness of headaches varied significantly by geography (χ² = 20.57, p = 0.005), with women in medically underserved areas less likely to consider their headaches serious. Women in rural areas were more likely to feel that physicians would not take their symptoms seriously (χ² = 5.99, p = 0.014). Race (χ² = 20.99, p = 0.021) and ethnicity (χ² = 24.15, p = 0.030) were associated with challenges related to insurance coverage. Higher HSAS scores were associated with increased physician-seeking behavior, F(2, 153) = 10.95, p < 0.001, η² = 0.13. A longer duration of experiencing headaches was also correlated with an increased likelihood of seeking care (χ² = 19.28, p = 0.037).

Conclusions

Factors such as geography, ethnicity, and duration of illness significantly shape healthcare engagement for low-income women with headaches. Future interventions should focus on improving provider availability, reducing access barriers, and building trust within affected communities. Limitations of this pilot study include recruitment challenges and an overrepresentation of a university-affiliated population, which may limit the generalizability of findings. Future studies should aim to recruit a larger and more diverse sample to validate these findings and identify additional patterns in care-seeking behavior.

## Introduction

Headache disorders are among the most common neurological conditions and a leading cause of disability worldwide, particularly among women of reproductive age [1]. Globally, migraine ranks as the second leading cause of years lived with disability, affecting more than one billion people [2]. In the United States, over 39 million individuals suffer from migraine, yet many remain undiagnosed or undertreated [3]. Headaches are generally categorized as either primary, such as migraine and tension-type headache, or secondary to another condition. While primary headaches represent the majority of clinical cases, they are often misunderstood or underestimated, especially outside specialized care settings [4].

The burden of headache is disproportionately high among low-income women [5], yet research on their care-seeking behaviors remains limited. Barriers to treatment in this population are multifactorial and include systemic and individual-level factors. Insurance status plays a critical role in access to care, with uninsured and publicly insured individuals reporting lower satisfaction with headache management, fewer consultations, and reduced access to pharmacologic and non-pharmacologic therapies [6]. Even when patients are insured, partial coverage for headache-related medications or visits can pose a significant financial burden, which contributes to delays in care or reliance on ineffective over-the-counter options [7].

Geographic disparities further compound barriers to headache care. Individuals living in rural and medically underserved regions often encounter limited availability of specialists, prolonged wait times, and substantial travel burdens, all of which may discourage care-seeking altogether [8]. The Appalachian population is particularly vulnerable, and within rural populations experiencing disparities, they are generally found to have worse health outcomes [9]. Transportation challenges represent a major obstacle in these settings, further limiting access to timely care [10]. In addition, language barriers and limited health literacy can impair patient-provider communication, which increases the risk of misdiagnosis, poor treatment adherence, and dissatisfaction with care [11].

Cultural stigma and internalized beliefs may also prevent women from seeking medical attention for headache symptoms [12]. Individuals may minimize the severity of their symptoms and perceive headache as a normal part of life rather than a legitimate medical issue. Patient distrust in the healthcare system or fear that their pain will not be taken seriously may also impair care-seeking behaviors. Women from racial or ethnic minority backgrounds are more likely to report feeling dismissed or unheard during clinical encounters [8].

Although previous studies have examined general barriers to headache care, there remains limited research exploring how individual perceptions and social determinants of health intersect to influence healthcare engagement among low-income women. Perceived symptom severity, cultural stigma, and mistrust in healthcare providers may shape whether individuals view their headaches as a condition warranting medical attention. These perceptions often interact with structural barriers such as insurance coverage and geographic access to create compounded obstacles to healthcare. By identifying associations between social, geographic, and psychological factors, this pilot study seeks to explore patterns of healthcare engagement among Medicaid-enrolled or uninsured women with recurrent headaches. In this study, healthcare engagement is defined as seeking clinical evaluation or treatment for headache symptoms from a physician or other healthcare professional.

This study was previously presented as a poster presentation at the 2025 Family Medicine Education Consortium (FMEC) Annual Meeting on September 20, 2025.

## Materials and methods

A cross-sectional survey was conducted in 2024 among 156 Medicaid-enrolled or uninsured women recruited through community-based outreach and Ohio University-affiliated email networks. Initial recruitment efforts involved in-person and virtual outreach, including flyer distribution across community spaces such as public libraries, family-owned grocery stores, and free clinics in Cleveland, Columbus, and Athens, Ohio. Due to limited participant recruitment through these methods, a secondary virtual recruitment strategy was implemented using a university-wide email distribution system at Ohio University. The survey was sent across all campuses, with recipients including students, faculty, and staff. Eligible participants were women aged 18 years or older who were either enrolled in state-sponsored Medicaid or uninsured. After eligibility screening, participants completed the Headache Severity and Affect Screening (HSAS) to confirm recurrent headache status and associated impact (see Appendix A). Participants who met HSAS eligibility thresholds were subsequently consented through an online consent form embedded within the survey. Following consent, participants completed survey items (see Appendix B) assessing demographics, geographic classification (rural, medically underserved area, or Appalachian), health literacy (measured using two questions evaluating the ability to interpret medical information and instructions), care-seeking behaviors, and barriers to accessing care. Participants who had previously sought physician care for headaches were asked to report satisfaction with care received and barriers encountered in obtaining optimal or ongoing treatment. Participants who had not sought physician care were asked questions regarding perceived obstacles to care, including stigma, transportation challenges, cost, availability of services, language barriers, mistrust in healthcare providers, and perceived headache severity. 

Instruments

Validated headache impact instruments are widely used in clinical and research settings to quantify symptom burden and functional impairment and were initially considered for participant eligibility assessment. However, because legal and licensing restrictions precluded the use of proprietary instruments in this study, an alternative screening measure was required. Consequently, the authors developed the HSAS questionnaire to assess perceived headache severity and functional impact in a manner suitable for use in a community-based survey.

Because the HSAS is a novel instrument, it was designed specifically for eligibility determination and exploratory severity stratification, rather than diagnostic classification. The HSAS was not intended to replace validated headache instruments; instead, it was developed to operationalize publicly available clinical guidance regarding when individuals with recurrent headaches should seek physician evaluation. Screening items assessed headache frequency, frequency of headache-related interference with daily activities, self-reported pain severity, participants’ ability to treat or avoid headaches, and perceived changes in headache patterns over time. Inclusion and exclusion criteria were informed by publicly available guidance from the Mayo Clinic and Cleveland Clinic regarding circumstances in which recurrent headaches warrant physician evaluation. Based on these criteria, HSAS items were categorized into tier 1, tier 2, and tier 3 questions. The HSAS has not yet undergone formal validation, and results using this measure should be interpreted as preliminary.

Tier 1 questions assessed the frequency with which headaches interfered with daily life, the severity of pain, and whether participants believed physician care could improve their quality of life. Responses indicating maximum functional impact, severe pain, and/or a perceived need for physician intervention resulted in immediate eligibility. Tier 2 questions assessed headache frequency, measured as days per month. Based on public-facing online educational resources from the Mayo Clinic and Cleveland Clinic, individuals experiencing headaches on 15 or more days per month are advised to seek medical evaluation, while those experiencing headaches on 8-15 days per month are advised to strongly consider physician care. Accordingly, participants reporting headaches on 15 or more days per month were automatically eligible, whereas those reporting 8-15 headache days per month were required to report at least moderate pain or functional impact to qualify. Tier 3 questions assessed relief from over-the-counter medications, awareness of headache triggers, and changes in headache patterns over time. These items allowed inclusion of participants who had attempted symptom management or experienced worsening headaches but may not have met tier 1 or tier 2 criteria alone.

Total HSAS scores ranged from 4 to 48 points. Study eligibility was defined as a total HSAS score of 12 or greater, with automatic inclusion for participants reporting a headache frequency of 15 or more days per month, regardless of total score. This frequency threshold alone is considered sufficient to warrant physician evaluation based on public-facing clinical guidance, independent of reported pain severity or functional impairment [13,14]. In addition, participants were automatically eligible if they reported responses indicating severe pain, constant interference with daily activities, or an explicit desire for physician-guided headache management, irrespective of the total HSAS score. Only participants meeting these HSAS eligibility criteria were included in the final analytic dataset.

Data analysis

All statistical analyses were conducted using Statistical Product and Service Solutions (SPSS, version 29.0.1.0; IBM SPSS Statistics for Windows, Armonk, NY). Descriptive and inferential statistics were performed to evaluate associations between demographic, geographic, and behavioral variables related to headache experiences and healthcare-seeking patterns. Pearson's chi-square and likelihood ratio tests were used to analyze categorical variables, while continuous variables (such as health literacy and headache screening scores) were compared using one-way analysis of variance (ANOVA). General linear models were used to evaluate the interaction effects between demographic factors.

Physician-seeking behavior (PHYS) and treatment-seeking behavior (TSB) were examined across demographic variables, including age group (AGRP), race (RACE), ethnicity (ETH), education level (EDU), and health literacy summary score (HLSUM). Separate analyses were conducted for the geographic influences of the Appalachian region (APP), rurality (RUR), and medically underserved area (MUA).

## Results

The survey identified several social and demographic factors associated with healthcare engagement among the 156 participants (Table 1).

**Table 1:** Social and Demographic Factors Associated With Healthcare Engagement *Race and ethnicity categories were not mutually exclusive; participants selecting multiple categories were counted in each applicable category. Percentages represent the proportion of participants within each category who did or did not seek care.

Characteristic	Group	Treatment Sought n (%)	No Treatment Sought n (%)
Insurance Status	Medicaid	81 (66.4%)	41 (33.6%)
	Uninsured	21 (61.8%)	13 (38.2%)
Race*	American Indian or Alaska Native	3 (75.0%)	1 (25.0%)
	Asian	7 (70.0%)	3 (30.0%)
	Black or African American	10 (83.3%)	2 (16.7%)
	Hispanic or Latino	4 (80.0%)	1 (20.0%)
	Native Hawaiian or Other Pacific Islander	1 (100.0%)	0 (0.0%)
	White	90 (75.0%)	30 (25.0%)
	Other	1 (100.0%)	0 (0.0%)
Ethnicity*	Middle Eastern/North African	3 (75.0%)	1 (25.0%)
	Asian	5 (83.3%)	1 (16.7%)
	European/White	68 (64.8%)	37 (35.2%)
	Hispanic/Latino	6 (75.0%)	2 (25.0%)
	African American	9 (69.2%)	4 (30.8%)
	Native Hawaiian or Pacific Islander	2 (100.0%)	0 (0.0%)
	American Indian or Alaska Native	2 (100.0%)	0 (0.0%)
	Other	5 (71.4%)	2 (28.6%)
Rural Residence	Yes	65 (73.9%)	23 (26.1%)
	No	37 (54.4%)	31 (45.6%)
Appalachian Region	Yes	64 (74.4%)	22 (25.6%)
	No	38 (54.3%)	32 (45.7%)
Medically Underserved Area (MUA)	Yes	30 (75.0%)	10 (25.0%)
	No	72 (62.1%)	44 (37.9%)
Education Level	Some high school	2 (66.7%)	1 (33.3%)
	High school diploma/GED	59 (67.0%)	29 (33.0%)
	Associate degree	23 (71.9%)	9 (28.1%)
	Bachelor degree	12 (60.0%)	8 (40.0%)
	Master’s degree or higher	6 (46.2%)	7 (53.8%)

**Figure 1:**
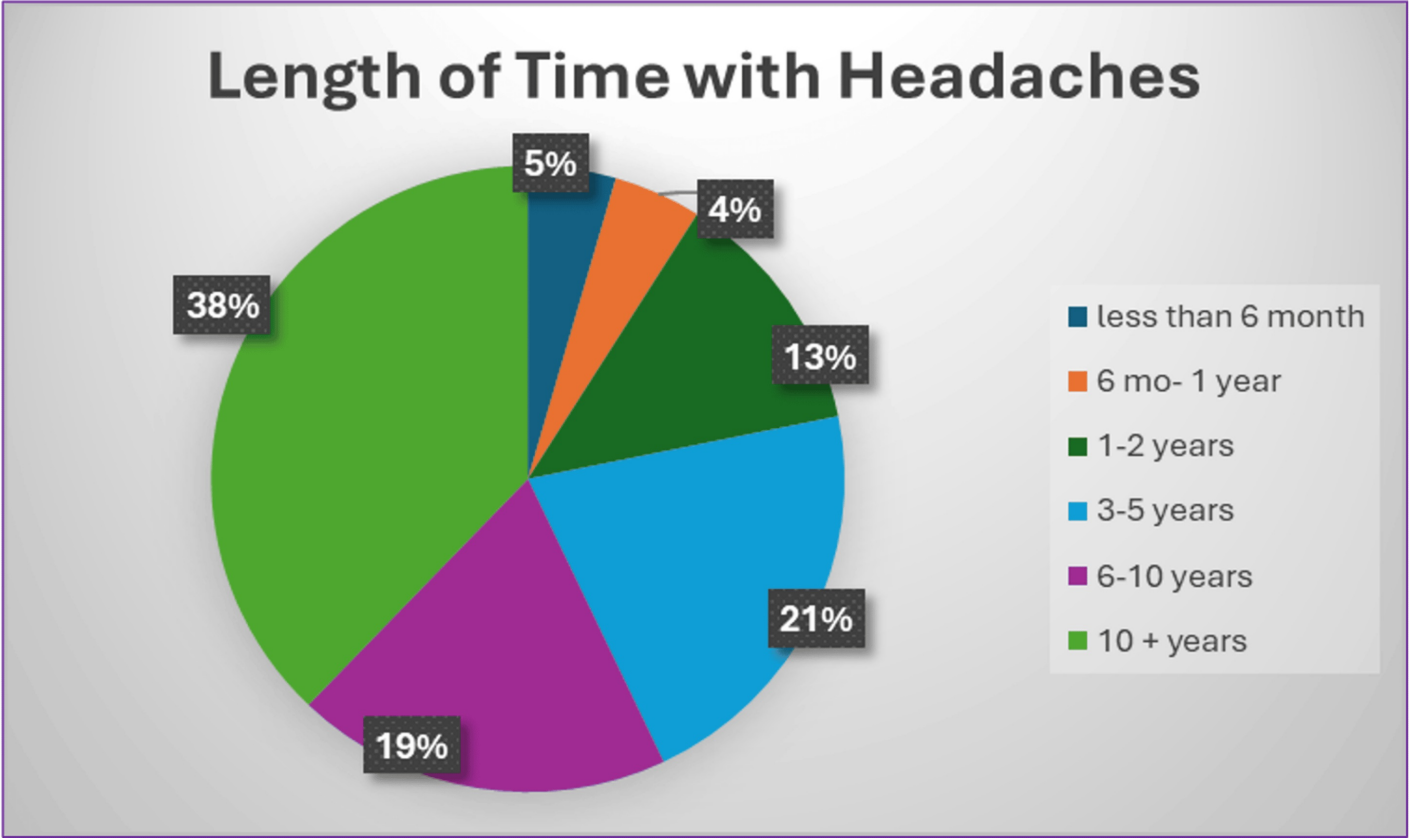
Length of Time With Headaches

Perceived headache seriousness differed significantly by geographic classification (χ² = 20.57, p = 0.005), with women residing in medically underserved areas being less likely to view their headaches as serious compared with those living in rural or Appalachian regions. Women in rural areas were also more likely to report concern that their headaches would not be taken seriously by a physician (χ² = 5.99, p = 0.014). Financial barriers to care varied by demographic characteristics. For example, race (χ² = 20.99, p = 0.021) and ethnicity (χ² = 24.15, p = 0.030) were significantly associated with reported challenges related to insurance coverage. Higher HSAS scores were significantly associated with greater physician-seeking behavior (F(2, 153) = 10.95, p < 0.001, η² = 0.13). Additionally, longer duration of headache experience was significantly associated with an increased likelihood of seeking care from a physician (χ² = 19.28, p = 0.037); notably, the majority of respondents reported experiencing headaches for more than 10 years (Figure 1).

Overall, headaches had a substantial impact on daily functioning, with 148 participants (95%) reporting interference with daily activities. Among the 98 participants who reported receiving care for headaches, 63% indicated satisfaction with their current treatment. Despite this, headache severity remained high, with 40% of participants rating their pain as severe or very severe, and only 19% reporting adequate relief with over-the-counter medications (Table 2).

**Table 2:** Survey Responses Related to Headache Frequency, Severity, and Coping

Survey Question	Response		N	Percent
How many days per month do you experience headaches?		0-7	69	44.2
How many days per month do you experience headaches?		8-14	66	42.3
How many days per month do you experience headaches?		15 plus	21	13.5
How often do headaches impact your ability to do your daily activities (household work, work, school, or social activities)?		Rare	8	5.1
How often do headaches impact your ability to do your daily activities (household work, work, school, or social activities)?		Sometimes	76	48.7
How often do headaches impact your ability to do your daily activities (household work, work, school, or social activities)?		Often	59	37.8
How often do headaches impact your ability to do your daily activities (household work, work, school, or social activities)?		Always	13	8.3
When you have a headache, how would you describe the pain?		Mild	12	7.7
When you have a headache, how would you describe the pain?		Moderate	81	51.9
When you have a headache, how would you describe the pain?		Severe	56	35.9
When you have a headache, how would you describe the pain?		Very severe	7	4.5
Would your life feel easier if a healthcare professional helped to manage your headaches?		Yes	95	60.9
Would your life feel easier if a healthcare professional helped to manage your headaches?		No	4	2.8
Would your life feel easier if a healthcare professional helped to manage your headaches?		Maybe	57	36.5
Do you find relief with over-the-counter medications?		Yes	30	19.2
Do you find relief with over-the-counter medications?		Sometimes	99	63.5
Do you find relief with over-the-counter medications?		No	27	17.3
Have your headaches gotten worse over time?		Yes	105	67.3
Have your headaches gotten worse over time?		No	22	14.1
Have your headaches gotten worse over time?		Unsure	29	18.6
Have you been able to identify what triggers your headaches?		Yes	10	6.4
Have you been able to identify what triggers your headaches?		Some	121	77.6
Have you been able to identify what triggers your headaches?		No	25	16.0

## Discussion

Our pilot survey identified several social and demographic factors associated with healthcare engagement among low-income women experiencing headaches. Perceived headache seriousness varied by geographic context, with women residing in medically underserved areas (MUAs) being less likely to view their headaches as serious compared with those living in rural or Appalachian regions. Women in rural areas were also more likely to report concern that their headaches would not be taken seriously by a physician. Financial barriers to care emerged across demographic groups, with race and ethnicity significantly associated with challenges related to insurance coverage. In addition, higher headache screening scores were associated with significantly increased physician-seeking behavior. Finally, a longer duration of headache experience was significantly associated with a greater likelihood of seeking care from a physician.

An important limitation of this study is the modest sample size. Participant recruitment proved challenging during the initial phases, as the study was designed to enroll a larger sample with more balanced representation across rural, medically underserved, and Appalachian populations. Initial recruitment efforts included flyer distribution at community locations, such as places of worship, public libraries, and local grocery stores; however, these approaches yielded limited participation. Consequently, recruitment largely relied on a university-wide email distribution system at Ohio University. This approach increased enrollment but resulted in a sample largely connected to academic environments. This recruitment approach may have introduced selection bias by overrepresenting individuals with higher baseline health literacy, greater access to health information, or increased familiarity with healthcare systems compared with the broader population of low-income women, thereby limiting generalizability. Despite this limitation, the sample retained variability in age and geographic background, and the observed associations highlight meaningful patterns in symptom perception, access barriers, and care-seeking behavior. Additionally, because the HSAS is a newly developed instrument with limited reported validation, it may introduce measurement bias. Future studies should formally validate the HSAS through reliability testing, assessment of construct validity, and correlation with established headache disability measures (e.g., HIT-6 or MIDAS).

The findings of this survey are largely consistent with existing literature on disparities in headache care. In Rhudy et al., examining emergency department (ED) utilization for migraine among rural and nonrural populations in Kentucky, the Appalachian subgroup demonstrated the highest rates of ED use, exceeding those of both rural and nonrural counterparts [9]. The authors attributed these differences to factors such as limited access to primary and neurologic specialty care, lower socioeconomic status, and higher opioid use within the Appalachian population [9]. Although our study examined symptom perception rather than ED utilization, both studies highlight meaningful geographic differences in how headache care is accessed and experienced across underserved populations.

Additionally, there is a broad consensus within the migraine and headache literature that insurance status is strongly associated with headache prevalence, care access, and outcomes. Uninsured individuals, Medicaid recipients, and those with low income demonstrate a higher prevalence of migraine compared with individuals holding private insurance [8]. Although overall headache and migraine prevalence is relatively similar across White, African American, and Hispanic populations in the United States, substantial disparities exist in care delivery and outcomes [8]. African American patients are more likely to report more frequent headaches and greater pain intensity, yet, even after adjustment for socioeconomic status, are less likely to receive a headache diagnosis or be prescribed acute migraine medications compared with White patients [8]. Individuals who experience both low socioeconomic status and racial or ethnic marginalization are therefore at increased risk for compounded health disparities. These findings align with our results demonstrating that race and ethnicity are significantly associated with insurance-related barriers to headache care.

As a pilot study, these findings provide valuable preliminary insight into the multifactorial barriers faced by low-income women with recurrent headaches and underscore the need for future studies.

## Conclusions

This pilot study highlights observed associations between social, geographic, and structural factors and healthcare engagement among low-income women experiencing headaches. Rather than reflecting a single barrier, patterns of healthcare engagement in this sample were associated with participants’ symptom perceptions, reported access constraints, insurance-related challenges, and prior healthcare experiences. These findings suggest that both individual perceptions and systemic factors may be relevant when considering disparities in headache-related care seeking. Future research should build on these preliminary findings using larger and more diverse samples to improve generalizability and enable more robust subgroup analyses. In particular, further investigation into headache prevalence, care access, and outcomes within Appalachian communities, with attention to region-specific challenges such as opioid use disorder and limited specialty care, is needed. Additional studies comparing urban medically underserved populations with rural counterparts may also clarify how geography and insurance status interact to influence disease burden and access to care. Moreover, expanded headache and migraine research focused on underrepresented racial and ethnic groups is critical to advancing equity in headache care for underserved women.

## Appendices

Appendix A. Headache Screening and Severity Score (HSAS)

Q1: How many days per month do you experience headaches? 

0-7 days: 1 point

8-15 days: 5 points

≥15 days: 9 points

Q2: How often do headaches impact your ability to do your daily activities (household work, work, school, or social activities)? 

Rarely: 0 points

Sometimes: 4 points

Often: 8 points

Always: 10 points

Q3: When you have a headache, how would you describe the pain? 

Mild: 0 points

Moderate: 4 points

Severe: 8 points

Very severe: 10 points

Q4: Would your life feel easier if a healthcare professional helped to manage your headaches? 

No: 0 points

Maybe: 8 points

Yes: 10 points

Q5: Do you find relief with over-the-counter medications? 

Yes: 1 point

Sometimes: 2 points

No: 3 points

Q6: Have your headaches gotten worse over time? 

No, they’ve stayed the same: 1 point

Not sure: 1 point

Yes, they’ve gotten worse: 3 points

Q7: Have you been able to identify what triggers your headaches? 

Yes, they are predictable: 1 point

I know some triggers, but not all of them: 2 points

No, they are unpredictable: 3 points

Appendix B: Survey Instrument

Q1: Race (select all that apply)

American Indian or Alaska Native

Asian

Black or African American

Hispanic or Latino

Native Hawaiian or Pacific Islander

White

Other
 

Q2: Ethnicity (select all that apply)

Middle Eastern/North African

Asian

European/White

Hispanic/Latino

African American

Native Hawaiian or Pacific Islander

American Indian or Alaska Native

Other 

Q3: ZIP Code

Q4: Highest Level of Education

Some high school

High school diploma or GED

Associate degree

Bachelor’s degree

Master’s degree or higher

Q5: Household Size (including yourself)
 

Q6: How often do you require assistance when reading instructions, pamphlets, or other written materials from a doctor or pharmacy?

Always

Sometimes

Never
 

Q7: How confident are you in filling out medical forms without assistance?

Extremely confident

Quite confident

Somewhat confident

A little confident

Not at all confident
 

Q8: How long have you been experiencing headaches?

Less than 6 months

6 months to 1 year

1-2 years

3-5 years

6-10 years

More than 10 years
 

Q9: Have you discussed your headaches with a physician?

Yes, primary care physician

Yes, neurologist or other specialist

No
 

Questions Only For Participants Who Have Sought Headache Care from a Physician

Q10: Are you satisfied with the care you are receiving?

Yes

No
 

Q11: Did you face any challenges while receiving care? (select all that apply)

Insurance did not cover appointment costs

Insurance did not cover medication costs

Difficulty scheduling appointments

Lack of transportation

Long wait times for appointments

Felt headaches were not taken seriously

Concern about language barriers

No challenges experienced

Please rank the following challenges from greatest to least impact:

Insurance coverage for appointments

Insurance coverage for medications

Scheduling difficulties

Transportation barriers

Long appointment wait times

Feeling not taken seriously by physicians

Language barriers

No challenges
 

Questions Only For Participants Who Have Not Sought Headache Care from a Physician

Q12: Why have you not seen a doctor for your headaches? (rank from greatest to least applicable)

Scheduling difficulties

Long wait times

Headaches not perceived as serious

Belief that a doctor cannot help

Concern about not being taken seriously

Insurance coverage for medications

Insurance coverage for appointments

Lack of health insurance

Transportation barriers

Language barriers

Stigma from social networks

Successful self-treatment

Other
 

Q13: Would you be more likely to visit a doctor if virtual appointments were available?

Yes

No

Not sure
